# Sensitivity Enhancement of Group Refractive Index Biosensor through Ring-Down Interferograms of Microring Resonator

**DOI:** 10.3390/mi13060922

**Published:** 2022-06-10

**Authors:** Hsuan Lai, Tzu-Ning Kuo, Jia-Yi Xu, Shih-Hsiang Hsu, Yi-Cheng Hsu

**Affiliations:** 1 Department of Electronic Engineering, National Taiwan University of Science and Technology, No. 43, Sec. 4, Keelung Rd., Taipei 10607, Taiwan; m10902337@mail.ntust.edu.tw (H.L.); m11002347@mail.ntust.edu.tw (T.-N.K.); m11002343@mail.ntust.edu.tw (J.-Y.X.); 2 Department of Biomechatronics Engineering, National Pingtung University of Science and Technology, 1 Shuefu Rd., Neipu 91201, Pingtung, Taiwan

**Keywords:** microring resonator, interferogram, biosensor, silicon photonics

## Abstract

In recent years, silicon-on-insulator substrates have been utilized for high-speed and low-power electronic components. Because of the high refractive index contrast of the silicon wire, its photonic device footprint can be significantly reduced. Moreover, the silicon photonic process is compatible with a complementary metal-oxide-semiconductor fabrication, which will benefit the high-density optoelectronic integrated circuits development. Researchers have recently proposed using the microring resonator (MRR) for label-free biosensing applications. The high-quality factor caused by the substantial electric field enhancement within the ring makes the MRR a good candidate for biomolecule detection under low analyte concentration conditions. This paper proposes an MRR chip to be a biosensor on the silicon platform through the relative displacement between the spatial ring-down interferograms at various cladding layers. The higher-order ring-down of the spatial interference wave packet will enhance the biosensing sensitivity after optimizing the coupling, MRR length, and the optical source bandwidth at the fixed optical waveguide loss. Finally, a typical sensitivity of 642,000 nm per refractive index unit is demonstrated under 0.1 μW minimum optical power detection for an MRR with a 100 μm radius. Higher sensitivity can be executed by a narrow bandwidth and lower silicon wire propagation loss.

## 1. Introduction

Biophotonics research is rapidly growing and has become one of the major developed biomedical technologies. The moving average and autoregressive types of refractive index-based interferometric biosensors are dedicated to the Mach–Zehnder interferometer (MZI) and microring resonator (MRR) for finite and infinite impulse responses, respectively [1,2,3]. In MZI, the signal variation may not be accurately detectable because of the large slope from the maximum and minimum of the sine and cosine functions. MRR owns the multiple cavity transmission and demonstrates the high-quality Q factor. Silicon-on-insulator (SOI) has successfully demonstrated the high-speed response and low electric power consumption. Furthermore, SOI fabrication is compatible with the well-established and mature complementary metal-oxide-semiconductor (CMOS) processing. Due to the unique property of the high index contrast between the core and cladding layers for a small footprint, SOI-based silicon wire waveguides were recently utilized as photonic biosensors [4,5,6].

Optical waveguide refractive index sensing has attracted considerable attention due to the waveguide’s immunity to electromagnetic interference, good compactness, robustness, and high compatibility with fiber networks while exhibiting shorter response time and higher sensitivities. Typically, the fluorescent material is labeled on the biomarkers to be characterized, but this method is time-consuming and costs considerably. Recently, researchers have proposed using the MRR for label-free biosensing applications [7,8], indicating the commercialization potential for the low cost, high yield, and portable biosensing platform to leverage CMOS processes. The substantial electric field enhancement within the ring makes the MRR illustrate the high Q factor and become a good candidate for cladding refractive index detection [9,10,11,12,13]. In silicon-based all-optical MRR biosensors, the spectral shifts from whispering gallery modes were applied to the single, multiplexer, and integrated platform of MRR. Another type of merging the optics and electrochemistry was utilized as multi-functional sensors through the n-doped silicon-based MRR and other cascaded structures [7]. All the above is spectral-shift-based MRR detection. A spatial domain ring-down of MRR is proposed in this paper for compatible biosensing sensitivity.

Refractive index sensors usually use optical power- and wavelength-related approaches for biosensing. Light power fluctuation may seriously affect the optical intensity sensitivity, and a cost-related high-resolution optical spectrum analyzer will limit the sensitivity from the acquired wavelengths. An MRR effective length, derived from the interferogram characterization, could also successfully demonstrate the sensing sensitivity.

Moreover, the round-trip ring-down waveforms will significantly enhance the sensitivity in the higher-order interferograms, which could be optimized and controlled by an optical waveguide propagation loss, coupling coefficient, and the input light bandwidth. To our best knowledge, the MRR effective length in the round-trip is the first to be illustrated as a group refractive index biosensor to enhance its sensitivity dramatically through the higher orders.

## 2. Theory and Design

**Figure 1:**
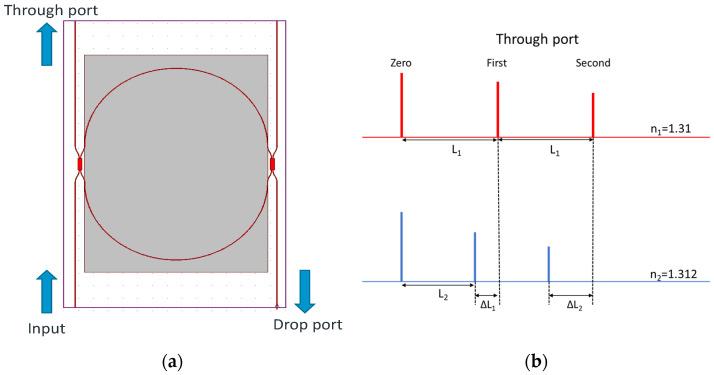
(**a**) MMI-coupled MRR with through and drop ports. (**b**) MRR interferograms.

A schematic of the multimode interference (MMI)-coupled MRR with the through and drop ports is illustrated in Figure 1a. The power transmission and *Q* factor in the through port waveguide are expressed in the following [14,15,16]:

(1) $${\left|T\right|}^{2}=\alpha_{MMI}{}^{2}\left[\frac{\alpha^{2}-2\alpha t\cos\theta +t^{2}}{1-2\alpha t\cos\theta +\alpha^{2}t^{2}}\right]$$

(2) $$Q=\frac{\lambda }{\Delta \lambda_{FWHM}}=\frac{2\pi n_{g}}{\alpha_{scat}\lambda }$$

where *λ* is the wavelength in vacuum, Δ*λ_FWHM_* is the resonant wavelength with the full width at half maximum (FWHM), *n_g_* is the ring waveguide group index, *α_scat_* is the magnitude of the optical scattering coefficient, *t*^2^ is the coupler power self-coupling coefficient, *α*^2^ is the power loss factor which includes both the ring loss and the coupler loss, *α*^2^ = *α_MMI_*^2^*α_ring_*^2^, and *θ* is the round trip phase accumulation and can be represented as 2*πn_g_L*/*λ* using the MRR perimeter length *L*.

The quality factor *Q* of optical waveguides is determined by intrinsic radiative losses, scattering losses, surface contaminants, and material losses [15]. The material loss-related *Q* can be written in Equation (2). In MRR, only the lower propagation loss in the waveguide can achieve a higher *Q* value. In Equation (1), the highest *Q* could be achieved through the minimum transmission, which can be resolved when the first derivative of Equation (1) is assumed as zero. Then, we can illustrate α to be equal to *t*, the condition for the MRR critical coupling.

The MRR interferograms under two ambient substances with refractive indices, *n*_1_ and *n*_2_, are shown in Figure 1b. The group indices for the strip silicon wire with 0.5 μm width and 0.22 μm height under the *n*_1_ and *n*_2_ cladding layers are represented as *n*_1*g*_ and *n*_1*g*_, respectively. Since the first ring-down waveform goes directly through the straight waveguide instead of the ring length, the first interferogram is called zero-order. Then, the following waveforms will keep going through the MRR with a round-trip type, and the sequential numbers will be applied to a succession of interferograms. When the *n*_2_ (1.312) is more extensive than *n*_1_ (1.31) for the cladding layers, *n*_1*g*_ is larger than *n*_2*g*_. Therefore, in the *n*_1_ cladding situation, the waveform distance, *L*_1_, between zero and the first order is the MRR perimeter length L multiplied by the group index, *n*_1*g*_, the same as the distance between the first and second waveforms. When the cladding layer is changed to *n*_2_, the waveform distance between zero and the first order becomes *L*_2_, equal to *L* multiplied by *n*_2*g*_. Since *n*_2*g*_ is more diminutive than *n*_1*g*_, the difference between *L*_1_ and *L*_2_ is Δ*L*_1_, written as around (*n*_1*g*_ − *n*_2*g*_)*L*. On the same principle, the first and second orders between *n*_1_ and *n*_2_ ambient substances, Δ*L*_2,_ can be represented as around 2*L*(*n*_1*g*_ − *n*_2*g*_). When the interferogram order is higher, the adjacent order distance difference will get larger to enhance the biosensing sensitivity enormously. However, the optical loss of the silicon wire and input light source bandwidth will play crucial roles in the waveform order number limitation. Therefore, the biosensing sensitivity optimization will be further discussed and demonstrated for the MRR-based ring-down interferograms. If various analyte concentrations are applied to the MRR length, the detectable waveforms with the most significant order number will experience the most extended shift for the highest sensitivity due to round-trip-based MRR.

The optical fiber was taken as the ring-down from the double-looped Mach–Zehnder interferometer for the refractive index sensor study [17,18]. In this paper, we propose to illustrate the higher-order interferograms from one MRR ring-down-based waveform in the spatial domain to enhance the sensitivity in refractive-index sensing. Due to the infinite impulse responses from MRR, the guiding light will travel the ring perimeter and form an interferogram. More waveforms in the spatial domain can be detected if the optical loss is low. The distance between two interferograms is the MRR perimeter length multiplied by the waveguide group index, which can be treated as the MRR effective length, *L*_eff_.

## 3. Results and Discussions

**Figure 2:**
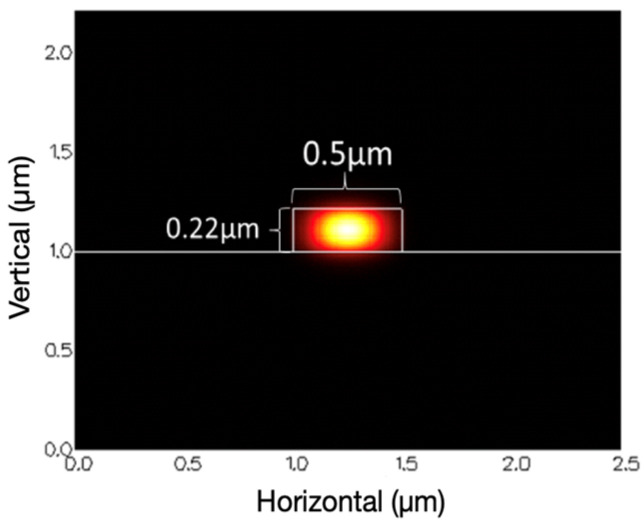
Silicon wire waveguide structure.

The silicon wire waveguide mode was calculated by a finite difference method (FDM) solver from the commercial software, Photon Design, shown in Figure 2. From Equation (2), the MRR group index was simulated at various cladding layers with different refractive indices, 1.31, 1.312, 1.314, 1.316, and 1.318. In Table 1, the silicon wire group index decreased when the refractive index of the surrounding detected substance increased.

**Figure 3:**
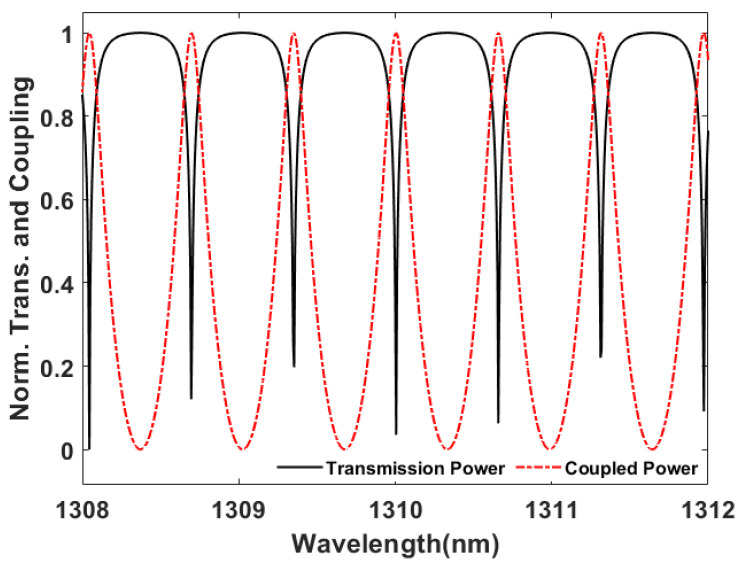
MRR spectrum from through and drop ports.

**Figure 4:**
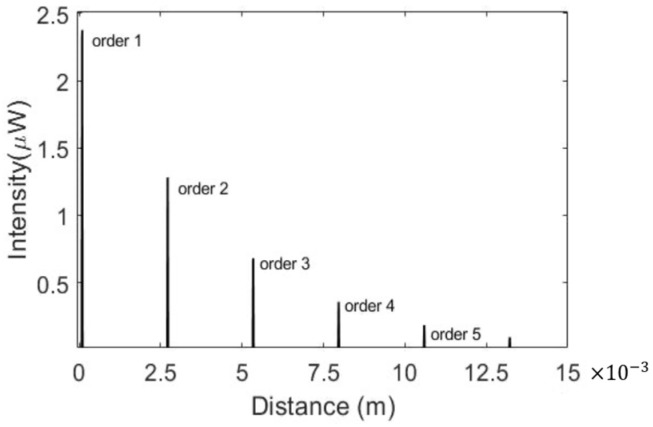
Ring-down waveforms of MRR.

The PICWave software from Photon Design was used to design and illustrate the performance of photonic integrated components. The optical model features of the 2D/3D electromagnetic simulator and time-domain travelling-wave model (TDTM) could faithfully predict the MRR spectrum and ring-down waveforms, respectively, to be shown in Figure 3 and Figure 4. Since the typical propagation loss of the optical waveguide from the multi-project wafer (MPW)-based foundries is around 2 dB/cm. The MRR perimeter length is treated as the circumference of the circle. The transmission power of the through port and coupling intensity of the drop port for a 100 μm radius-based MRR are all shown in Figure 3. The *Q* value from them through the port is 55,274. Then the spatial domain from the 43.75 fs optical pulse width with 22.86 μW power at 1310 nm wavelength through TDTM of PICWave could be derived to form the ring-down based waveforms, shown in Figure 4. When the optical loss is low, more order interferograms could be demonstrated. In this paper, 0.1 μW is taken as the optical power detection limit for all the performance discussions. Therefore, only six waveform orders are shown in Figure 4.

The typical resonant wavelength-based sensitivity for a microring resonator (MRR) is defined as *S_λ_* = ∆*λ*/∆*n*_clad_, where ∆*λ* and ∆*n*_clad_ represent the resonant wavelength peak shift and the refractive index change of the cladding material, respectively [4]. In this paper, the ring-down based MRR is time-domain interferograms, and the spatial sensitivity is defined as

$$S_{L}=\frac{\Delta L}{\Delta n_{g}}$$

where ∆*L* is the interference waveform shifting, and ∆*n_g_* is the group index variation of the cladding materials.

In Table 2, further study will be demonstrated in the adjacent order shift distance and sensitivity for five different MRR radii, 50, 75, 100, 125, and 150 μm, under two cladding-layer refractive indices 1.31 and 1.312. Under the 0.1 μW detection limit for the waveform, the larger radius can form a long adjacent interferogram order, but the detectable order number is decreased. Therefore, when the typical silicon wire propagation loss, 2 dB/cm, of MPW is considered, MRR with a 100 μm radius can demonstrate the highest sensitivity 642,000 nm/RIU, where RIU denotes the refractive index unit.

**Figure 5:**
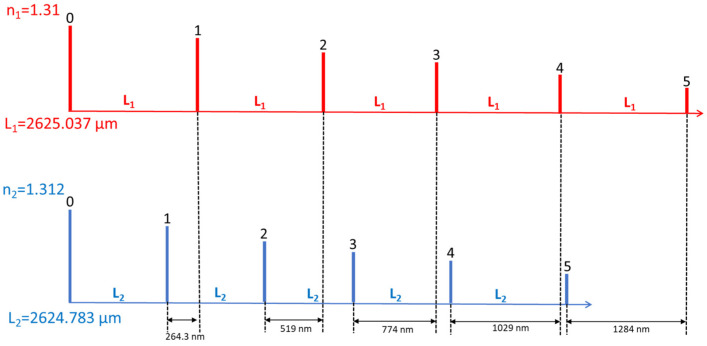
The different interferogram orders in the spatial domain for the MRR with a 100 μm radius.

In Figure 5, the interferograms from the MRR with a 100 μm radius demonstrate five orders under two ambient substances, *n*_1_ (1.31) and *n*_2_ (1.312). The waveform of order 3 and order 2 are two and three times more than order 1. The same principle can be applied to the higher order. L_1_ is the MRR perimeter length multiplied by the waveguide group index *n_g_*_1_. Finally, a 1284 nm shift distance can be gauged and characterized by the optical low-coherence interferometry (OLCI). The OLCI comprises the Mach–Zehnder structure, low coherence light source, and step motor stage [19].

**Figure 6:**
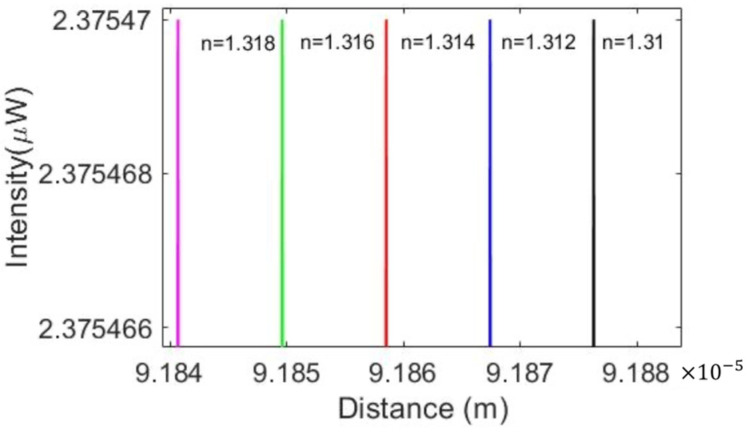
The first order comparison among five different refractive indices.

Five refractive indices, 1.31, 1.312, 1.314, 1.316, and 1.318, were applied to the cladding layers. The first order of the MRR with a 100 μm radius illustrates a lower effective length and shifts to the left due to the smaller group index after the ambient refractive index increased from 1.31 to 1.312. When the cladding layer refractive index keeps rising, the first order will shift to the left more, as shown in Figure 6. Similarly, the higher interferogram order will experience more shifts than the first order under the same cladding layer conditions.

**Figure 7:**
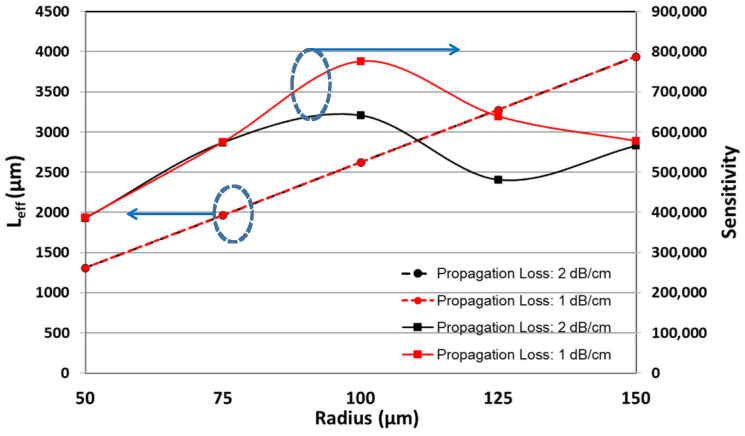
The comparison for the sensitivity and effective length between two propagation losses.

If the waveguide propagation loss can be improved to 1 dB/cm, the simulation shows the highest sensitivity is enhanced to 776,500 nm/RIU. The related simulation data are shown in Table 3. The comparison of the sensitivity and effective length between two propagation losses is shown in Figure 7. In Figure 7, two plotting versus various MRR radii are demonstrated at two propagation losses, 2 dB/cm and 1 B/cm. The propagation loss does not affect the effective length of the ring-down interferogram. However, the lower propagation loss, 1 dB/cm, shows higher sensitivity than 2 dB/cm after the radius is more extensive than 75 μm. The reason is that the highest waveform order, which can be minimally detected from ring-down MRR interferograms in the spatial domain, is prominent in the lower propagation loss.

**Figure 8:**
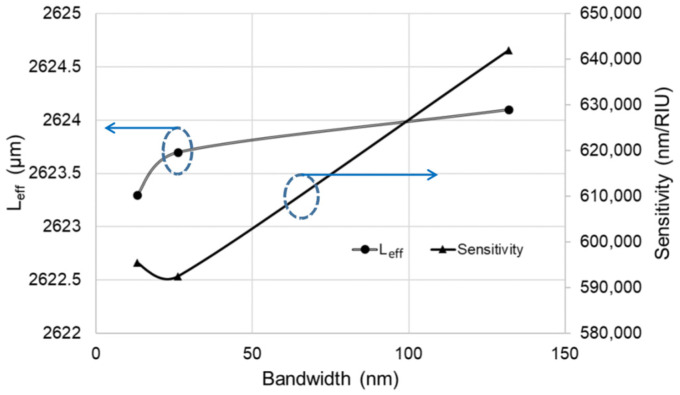
The comparison for the sensitivity and effective length among various bandwidths of input lights.

Since the optical pulse width will affect the ring-down order number, the input light bandwidths from TDTM were changed from 132 nm, 26.2 nm, and 13.2 nm. The ring-down order for a 132 nm bandwidth under the same 100 μm radius MRR and same pulse light with 22.86 μW power at 1310 nm wavelength could be detected more. In Table 4, the sensitivity shows 642,000 nm/RIU. When the input light bandwidth is less than 132 nm, the waveform order can be sensed less, and the sensitivity is decreased, as shown in Figure 8. We can also conclude that the bandwidth does not affect the ring-down interferogram’s effective length.

A 0.1 μW was taken as the optical power detection limit for the sensitivity study. Suppose the input optical power is enlarged or the photodetector is built with lower shot and dark noises. In that case, the highest order of the ring-down interferogram can be further detected to enhance the sensitivity. The critical coupling, α = t, from the MRR can obtain the most significant quality factor [13], and the MRR radius becomes 2396 μm. Then the sensitivity will be increased up to 7,605,000 nm/RIU from the 43.75 fs optical pulse width with 22.86 μW power at 1310 nm wavelength through TDTM.

The spatial resolution determines the limit of detection (LOD), either by the moving accuracy of motorized stages, 18.9 nm, or the optical ruler, 655 nm [19]. In this paper, the ring-down-based MRR demonstrates time-domain interferograms. The spatial sensitivity is defined as the interference waveform shifting divided by the group index variation of the cladding materials. When the non-critical coupling (100 μm radius) is applied, the LODs for the optical ruler and motorized stage are 1 × 10^−4^ (=655/642,000) RIU and 2.9 × 10^−5^ (=18.9/642,000) RIU, respectively, where 642,000 nm/RIU is the sensitivity from the ring-down interferogram of the MRR with 100 μm radius. If the critical coupling is taken as an example, the MRR radius should be 2396 μm. The LODs are 8.5 × 10^−5^ (=655/7,605,000) RIU and 2.48 × 10^−6^ (=18.9/7,605,000) RIU from the optical ruler and stepper motor, respectively, where 7,605,000 nm/RIU is the sensitivity from the ring-down interferogram of the MRR with 2396 μm radius.

## 4. Conclusions

The MRR is widely discussed for its small size, simple structure, and high sensitivity. The waveguide-based MRR is operated through the ring perimeter, refractive index, optical coupler, and propagation loss. The light injected into the MRR results in a particular period in the wavelength domain from the through port. A conventional MRR is mainly utilized to observe the change of resonant wavelengths through this feature. We proposed to optimize the MRR round-trip ring-down waveforms interrogated with the propagation loss and light pulse width to enhance the biosensing sensitivity dramatically through the higher interferogram orders. Moreover, the current photodetector detection limit is 0.1 μW. Lower optical power detection could highly increase the sensitivity by one or two orders.

## Figures and Tables

**Table 1:** Silicon wire group index variation under various cladding refractive indices.

Refractive Index	Group Index
1.31	4.177103
1.312	4.176699
1.314	4.176294
1.316	4.175889
1.318	4.175483

**Table 2:** The order shift distance and sensitivity for five different MRR radii under two cladding-layer refractive indices—1.31 and 1.312 for 2 dB/cm propagation loss. (R represents the radius.).

Propagation Loss: 2 dB/cm										
	R = 50		R = 75		R = 100		R = 125		R = 150	
Order	Shift Value (nm)	Sensitivity	Shift Value (nm)	Sensitivity	Shift Value (nm)	Sensitivity	Shift Value (nm)	Sensitivity	Shift Value (nm)	Sensitivity
1	135.3	67,650	198.6	99,300	264.3	132,150	327	163,500	390	195,000
2	261.9	130,950	390	195,000	519	259,500	645	322,500	771	385,000
3	387	193,500	576	288,000	774	387,000	963	481,500	1152	576,000
4	516	258,000	768	384,000	1029	514,500	NA	NA	NA	NA
5	642	321,000	957	478,500	1284	642,000	NA	NA	NA	NA
6	771	385,500	1149	574,500	NA	NA	NA	NA	NA	NA

**Table 3:** The order shift distance and sensitivity for five different MRR radii under two cladding-layer refractive indices—1.31 and 1.312 for 1 dB/cm propagation loss.

Propagation Loss: 1 dB/cm										
	R = 50		R = 75		R = 100		R = 125		R = 150	
Order	Shift Value (nm)	Sensitivity	Shift Value (nm)	Sensitivity	Shift Value (nm)	Sensitivity	Shift Value (nm)	Sensitivity	Shift Value (nm)	Sensitivity
1	135.3	67,650	199.7	99,850	264.8	132,400	327.6	163,800	392	196,000
2	262.8	131,400	391	195,500	521	260,500	647	323,500	774	387,000
3	387	193,500	577	288,500	780	390,000	965	482,500	1156	578,000
4	517	258,500	768.6	384,300	1026	513,000	1278.9	639,450	NA	NA
5	642	321,000	956	478,000	1290	645,000	NA	NA	NA	NA
6	774	387,000	1151	575,500	1553	776,500	NA	NA	NA	NA

**Table 4:** The bandwidth effect on the spatial sensitivity in the ring-down based MRR.

	Bandwidth = 132 nm		Bandwidth = 26.2 nm		Bandwidth = 13.2 nm	
Order	Shift Value (nm)	Sensitivity (nm/RIU)	Shift Value (nm)	Sensitivity (nm/RIU)	Shift Value (nm)	Sensitivity (nm/RIU)
1	264.3	132,150	423	211,500	432	216,000
2	519	259,500	681	340,500	684	342,000
3	774	387,000	933	466,500	939	469,500
4	1029	514,500	1185	592,500	1191	595,500
5	1284	642,000	NA	NA	NA	NA
6	NA	NA	NA	NA	NA	NA

## Data Availability

Not applicable.
