# Correction: Lai et al. Sensitivity Enhancement of Group Refractive Index Biosensor through Ring-Down Interferograms of Microring Resonator. *Micromachines* 2022, *13*, 922

**DOI:** 10.3390/mi13071152

**Published:** 2022-07-21

**Authors:** Hsuan Lai, Tzu-Ning Kuo, Jia-Yi Xu, Shih-Hsiang Hsu, Yi-Cheng Hsu

**Affiliations:** 1 Department of Electronic Engineering, National Taiwan University of Science and Technology, No. 43, Sec. 4, Keelung Rd., Taipei 10607, Taiwan; m10902337@mail.ntust.edu.tw (H.L.); m11002347@mail.ntust.edu.tw (T.-N.K.); m11002343@mail.ntust.edu.tw (J.-Y.X.); 2 Department of Biomechatronics Engineering, National Pingtung University of Science and Technology, 1 Shuefu Rd., Neipu 91201, Pingtung, Taiwan

In the original publication [1], there was a mistake in Table 3, as published. The content of Table 3 was repeated with Table 4. The corrected Table 3 appears below.

The authors apologize for any inconvenience caused and state that the scientific conclusions are unaffected. This correction was approved by the Academic Editor. The original publication has also been updated.

## Figures and Tables

**Table 3:** The order shift distance and sensitivity for five different MRR radii under two cladding-layer refractive indices—1.31 and 1.312 for 1 dB/cm propagation loss.

Propagation Loss: 1 dB/cm										
	R = 50		R = 75		R = 100		R = 125		R = 150	
Order	Shift Value (nm)	Sensitivity	Shift Value (nm)	Sensitivity	Shift Value (nm)	Sensitivity	Shift Value (nm)	Sensitivity	Shift Value (nm)	Sensitivity
1	135.3	67,650	199.7	99,850	264.8	132,400	327.6	163,800	392	196,000
2	262.8	131,400	391	195,500	521	260,500	647	323,500	774	387,000
3	387	193,500	577	288,500	780	390,000	965	482,500	1156	578,000
4	517	258,500	768.6	384,300	1026	513,000	1278.9	639,450	NA	NA
5	642	321,000	956	478,000	1290	645,000	NA	NA	NA	NA
6	774	387,000	1151	575,500	1553	776,500	NA	NA	NA	NA
